# (3*R*,4*R*,5*R*)-5-(Acetamido­meth­yl)-*N*-benzyl-3,4-dihy­droxy­tetra­hydro­furan-3-carboxamide

**DOI:** 10.1107/S1600536810039589

**Published:** 2010-10-09

**Authors:** Michela I. Simone, Alison A. Edwards, Andrew R. Cowley, George W. J. Fleet, David J. Watkin

**Affiliations:** a Chemistry Research Laboratory, University of, Oxford, Mansfield Road, Oxford, OX1 3TA, England

## Abstract

X-ray crystallographic analysis with Cu Kα radiation established the relative configurations of the stereogenic centers in the title compound, C_15_H_20_N_2_O_5_, and clarified mechanistic ambiguities in the synthesis. The conformation of the five-membered ring approximates twisted, about a C—O bond. The absolute configuration of this carbon-branched dipeptide isostere was known based on the use of d-ribose as the starting material. Refinement of the Flack parameter gave an ambiguous result but the refined Hooft parameter is in agreement with the assumed (d-ribose) absolute structure. The crystal structure consists of N—H⋯O and O—H⋯O hydrogen-bonded bi-layers, with the terminal methyl and phenyl groups forming a hydro­phobic inter-layer inter­face. Some weak C—H⋯O inter­actions are also present.

## Related literature

For reviews of sugar amino acids, see: Risseeuw *et al.* (2007 ▶); Smith & Fleet (1999 ▶). For investigations of peptidomimetics, see: Smith *et al.* (1998 ▶); Long *et al.* (1998 ▶, 1999 ▶); Claridge *et al.* (1999 ▶); Brittain *et al.* (2000 ▶); Hungerford *et al.* (2000 ▶); Raunkjr *et al.* (2004 ▶); Jockusch *et al.* (2006 ▶); Tuin *et al.* (2009 ▶). For crossed-aldol reactions of carbohydrates with formaldehyde, see: Ho (1985 ▶); Simone *et al.* (2005 ▶, 2008 ▶); Best *et al.* (2010 ▶). For more strategies for the synthesis of branched carbohydrates, see: Hotchkiss *et al.* (2004 ▶); Soengas *et al.* (2005 ▶); Booth *et al.* (2008 ▶). For a related structure, see: Punzo *et al.* (2005 ▶). For the treatment of hydrogen atoms in *CRYSTALS*, see: Cooper *et al.* (2010 ▶). For the determination of absolute configuration, see: Hooft *et al.* (2008 ▶).
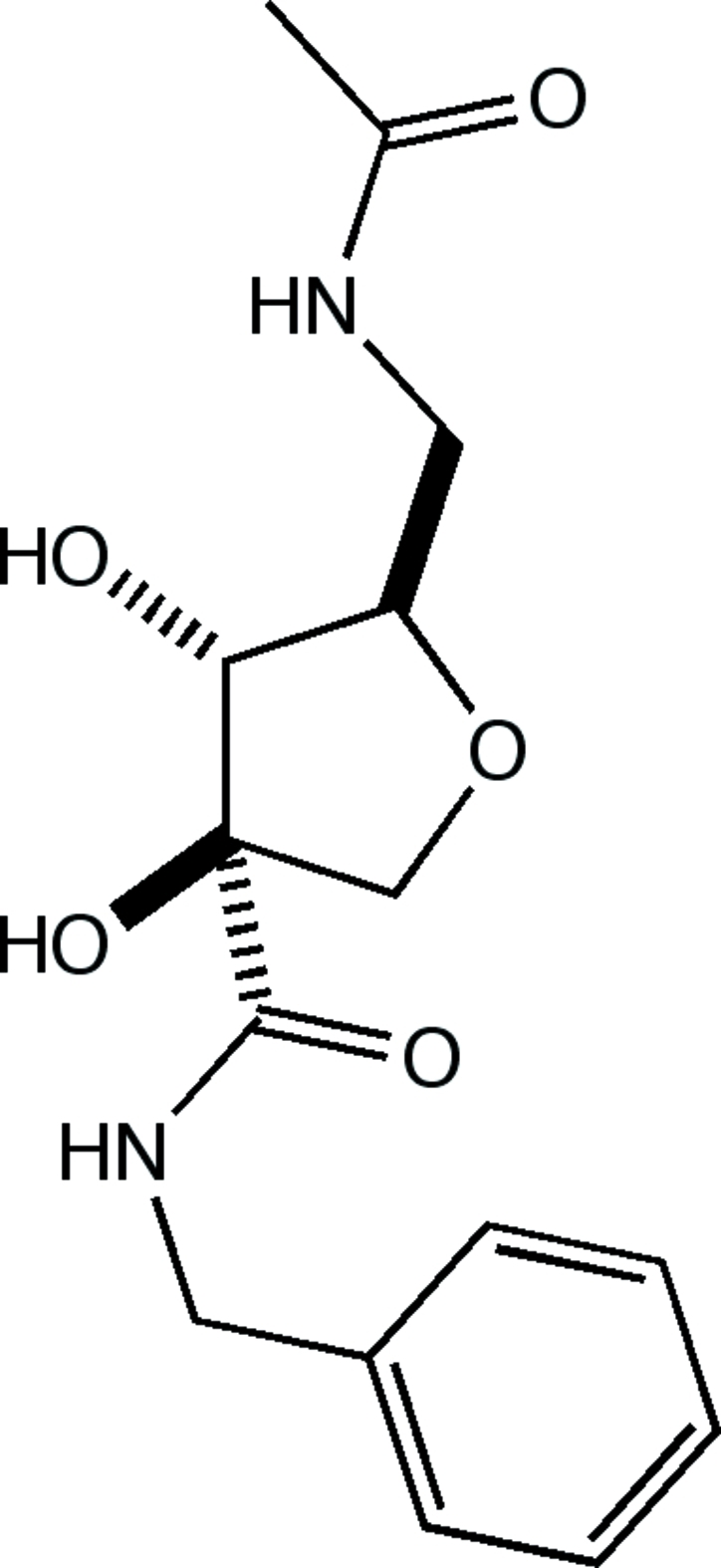


## Experimental

### 

#### Crystal data

C_15_H_20_N_2_O_5_
*M* *_r_* = 308.33
Orthorhombic, 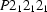

*a* = 5.4130 (2) Å
*b* = 8.5082 (2) Å
*c* = 32.6501 (4) Å
*V* = 1503.70 (7) Å^3^
*Z* = 4
Cu *K*α radiation
μ = 0.86 mm^−1^
*T* = 150 K
0.35 × 0.20 × 0.04 mm

#### Data collection

Oxford Diffraction Gemini diffractometer
Absorption correction: multi-scan (*CrysAlis RED*; Oxford Diffraction, 2002 ▶) *T*_min_ = 0.95, *T*_max_ = 0.97
12797 measured reflections
1805 independent reflections
1371 reflections with *I* > 2.0σ(*I*)
*R*_int_ = 0.038
θ_max_ = 54.3°

#### Refinement

*R*[*F* ^2^ > 2σ(*F* ^2^)] = 0.033
*wR*(*F* ^2^) = 0.061
*S* = 0.99
1796 reflections
200 parameters
H-atom parameters constrained
Δρ_max_ = 0.21 e Å^−3^
Δρ_min_ = −0.28 e Å^−3^
Absolute structure: Flack (1983 ▶), 681 Friedel pairs
Flack parameter: 0.0 (3)

### 

Data collection: *Gemini* (Oxford Diffraction, 2006 ▶); cell refinement: *CrysAlis RED* (Oxford Diffraction, 2002 ▶); data reduction: *CrysAlis RED*; program(s) used to solve structure: *SIR92* (Altomare *et al.*, 1994 ▶); program(s) used to refine structure: *CRYSTALS* (Betteridge *et al.*, 2003 ▶); molecular graphics: *CAMERON* (Watkin *et al.*, 1996 ▶); software used to prepare material for publication: *CRYSTALS*.

## Supplementary Material

Crystal structure: contains datablocks global, I. DOI: 10.1107/S1600536810039589/hb5639sup1.cif

Structure factors: contains datablocks I. DOI: 10.1107/S1600536810039589/hb5639Isup2.hkl

Additional supplementary materials:  crystallographic information; 3D view; checkCIF report

## Figures and Tables

**Table 1:** Hydrogen-bond geometry (Å, °)

*D*—H⋯*A*	*D*—H	H⋯*A*	*D*⋯*A*	*D*—H⋯*A*
O6—H61⋯O7^i^	0.80	1.95	2.737 (4)	167
O7—H71⋯O1^ii^	0.80	2.08	2.821 (4)	154
N10—H101⋯O21^i^	0.84	2.29	3.023 (4)	147
N19—H191⋯O9^iii^	0.88	2.04	2.861 (4)	155
C2—H22⋯O21^iii^	0.98	2.45	3.322 (4)	148
C4—H41⋯O1^iv^	0.99	2.59	3.520 (4)	156
C4—H41⋯O7^i^	0.99	2.49	3.257 (4)	134

Symmetry codes: (i) 
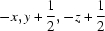
; (ii) 
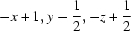
; (iii) 
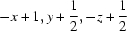
; (iv) 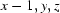.

**Fig. 1.**
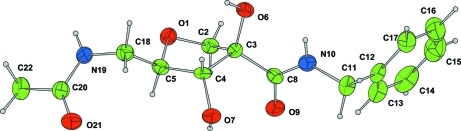
The title compound with displacement ellipsoids drawn at the 50% probability level. H atoms are shown as spheres of arbitary radius.

**Fig. 2.**
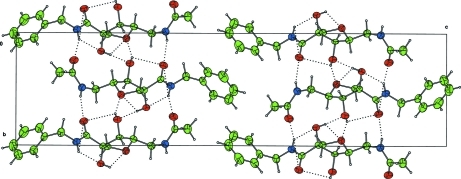
The hydrogen bonded bi-layer viewed along the 'a' axis. The hydrophobic faces to the layers explains why the thin platey crystals were extremely fragile.

**Fig. 3.**
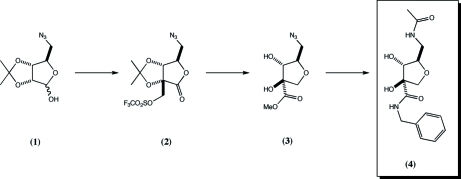
Preparation of the title compound.

## supplementary crystallographic information

### Comment

δ-Tetrahydrofuran sugar amino acids (THF SAAs) (Risseeuw *et al.*, 2007; Smith and Fleet, 1999) have been extensively used as dipeptide isosteres (Long *et al.*, 1998; Brittain *et al.*, 2000) and incorporated into peptidomimetics (Smith *et al.*, 1998; Hungerford *et al.*, 2000; Raunkjr *et al.*, 2004). The secondary structural preferences induced by THF SAAs upon their incorporation into peptidomimetics have been investigated in a wide variety of systems (Long *et al.*, 1999; Tuin *et al.*, 2009). All these studies have been reported on linear carbon chains, however a number of approaches to branched carbohydrates have been developed (Soengas *et al.*, 2005; Hotchkiss *et al.*, 2004; Booth *et al.*, 2008). This will allow the synthesis of THF SAAs with a branched carbon chain and allow a new family of foldamers to be created.

Compound (4) is a dipeptide isostere bearing a branching carbon substituent at position C-3 of the THF scaffold. The branched peptidomimetic (4) was synthesized in a number of steps from the suitably protected D-ribose derivative (1). A crossed aldol reaction of (1) with formaldehyde (Ho, 1985) gave the resulting lactol which was then further modified to give the carbon-branched lactone trifluoromethanesulfonate (2). Reaction of (2) in acidic methanol (Simone *et al.*, 2008) afforded the branched δ-THF SAA (3). The dipeptide isostere (4) was synthesized from SAA scaffold (3) in four synthetic steps. The crystal structure of an isomeric branched peptidomimetic has been published previously (Punzo *et al.*, 2005). The crystal structure of (4) removes mechanistic ambiguities arising from the Ho crossed aldol condensation and successive modifications of the THF scaffold. Furthermore the crystal structure may provide information about the conformational preference of the scaffold (4) and its corresponding ability to induce any secondary structural features when incorporated into peptidomimetics. The use of *D*-ribose as the starting material enabled the determination of the absolute configuration of this carbon-branched dipeptide isostere (4).

### Experimental

Compound (4) was dissolved in methanol, ethyl acetate and cyclohexane and then crystallized as the solvent (methanol) evaporated slowly to give crystals of (I) as very thin fragile colourless plates, which readily delaminated on handling. M.p. 428.2–429.2 K; [α]_D_^23^ +0.64 (c, 0.70 in methanol) (Simone *et al.*, 2005).

### Refinement

Refinement of the Flack (1983) parameter was inconclusive, 0.0 (3), but the Hooft parameter, 0.01 (6) was in agreement with the known absolute configuration (Hooft *et al.*, 2008).

The H atoms were all located in a difference map, but those attached to carbon atoms were repositioned geometrically. The H atoms were initially refined with soft restraints on the bond lengths and angles to regularize their geometry (C—H in the range 0.93–0.98, N—H = 0.86, O—H = 0.82 Å) and *U*_iso_(H) (in the range 1.2–1.5 times *U*_eq_ of the parent atom), after which the positions were refined with riding constraints (Cooper *et al.*, 2010).

### Crystal data

**Table:**

C_15_H_20_N_2_O_5_	*D*_x_ = 1.362 Mg m^−^^3^
*M**_r_* = 308.33	Cu *K*α radiation, λ = 1.54180 Å
Orthorhombic, *P*2_1_2_1_2_1_	Cell parameters from 6490 reflections
*a* = 5.4130 (2) Å	θ = 2–50°
*b* = 8.5082 (2) Å	µ = 0.86 mm^−^^1^
*c* = 32.6501 (4) Å	*T* = 150 K
*V* = 1503.70 (7) Å^3^	Plate, colourless
*Z* = 4	0.35 × 0.20 × 0.04 mm
*F*(000) = 656	

### Data collection

**Table:**

Oxford Diffraction Gemini diffractometer	1371 reflections with *I* > 2.0σ(*I*)
graphite	*R*_int_ = 0.038
ω scans	θ_max_ = 54.3°, θ_min_ = 5.4°
Absorption correction: multi-scan (*CrysAlis RED*; Oxford Diffraction, 2002)	*h* = −5→5
*T*_min_ = 0.95, *T*_max_ = 0.97	*k* = −8→8
12797 measured reflections	*l* = −33→34
1805 independent reflections	

### Refinement

**Table:**

Refinement on *F*^2^	Hydrogen site location: inferred from neighbouring sites
Least-squares matrix: full	H-atom parameters constrained
*R*[*F*^2^ > 2σ(*F*^2^)] = 0.033	Method = Modified Sheldrick *w* = 1/[σ^2^(*F*^2^) + ( 0.1*P*)^2^ + 0.0*P*] , where *P* = (max(*F*_o_^2^,0) + 2*F*_c_^2^)/3
*wR*(*F*^2^) = 0.061	(Δ/σ)_max_ = 0.0003
*S* = 0.99	Δρ_max_ = 0.21 e Å^−^^3^
1796 reflections	Δρ_min_ = −0.28 e Å^−^^3^
200 parameters	Absolute structure: Flack (1983), 681 Friedel pairs
0 restraints	Flack parameter: 0.0 (3)
Primary atom site location: structure-invariant direct methods	

### Special details

**Table:**

Experimental. The crystal was placed in the cold stream of an Oxford Cryosystems open-flow nitrogen cryostat (Cosier & Glazer, 1986) with a nominal stability of 0.1 K.Cosier, J. & Glazer, A.*M*., 1986. J. Appl. Cryst. 105 107.The 9 missing reflections are all low-angle and in the penumbra of the beam trap. Their indices are 1 0 1 0 1 1 0 1 2 0 1 3 0 0 4 0 1 4 0 1 5 0 0 6

### Fractional atomic coordinates and isotropic or equivalent isotropic displacement parameters (Å^2^)

**Table:**

	*x*	*y*	*z*	*U*_iso_*/*U*_eq_	
O1	0.5384 (3)	0.54901 (19)	0.24407 (4)	0.0310	
C2	0.5370 (4)	0.5114 (3)	0.28721 (7)	0.0285	
C3	0.2641 (4)	0.5070 (3)	0.29786 (7)	0.0260	
C4	0.1541 (4)	0.4286 (3)	0.25891 (7)	0.0254	
C5	0.3580 (4)	0.4433 (3)	0.22659 (7)	0.0292	
O6	0.1751 (3)	0.6640 (2)	0.30274 (4)	0.0326	
O7	0.0770 (3)	0.27045 (18)	0.26442 (4)	0.0315	
C8	0.2035 (5)	0.4123 (4)	0.33591 (8)	0.0282	
O9	0.3097 (3)	0.2865 (2)	0.34244 (5)	0.0385	
N10	0.0228 (4)	0.4687 (2)	0.35927 (6)	0.0333	
C11	−0.0761 (5)	0.3862 (3)	0.39501 (7)	0.0386	
C12	0.0572 (5)	0.4266 (4)	0.43394 (8)	0.0357	
C13	0.2683 (6)	0.3472 (4)	0.44544 (9)	0.0475	
C14	0.3884 (6)	0.3856 (4)	0.48131 (11)	0.0609	
C15	0.3030 (7)	0.5041 (5)	0.50564 (9)	0.0650	
C16	0.0949 (7)	0.5856 (4)	0.49430 (9)	0.0609	
C17	−0.0278 (5)	0.5475 (4)	0.45838 (8)	0.0465	
C18	0.2707 (4)	0.5031 (3)	0.18519 (7)	0.0335	
N19	0.4531 (4)	0.4865 (2)	0.15327 (6)	0.0334	
C20	0.4739 (5)	0.3543 (4)	0.13069 (8)	0.0327	
O21	0.3280 (4)	0.2440 (2)	0.13419 (5)	0.0418	
C22	0.6860 (5)	0.3518 (3)	0.10127 (8)	0.0482	
H61	0.1223	0.6945	0.2814	0.0479*	
H71	0.1969	0.2186	0.2694	0.0465*	
H101	−0.0317	0.5582	0.3538	0.0384*	
H191	0.5586	0.5638	0.1497	0.0387*	
H22	0.6302	0.5904	0.3029	0.0333*	
H21	0.6124	0.4078	0.2913	0.0332*	
H41	0.0066	0.4876	0.2499	0.0266*	
H51	0.4304	0.3372	0.2225	0.0326*	
H111	−0.2474	0.4142	0.3983	0.0447*	
H112	−0.0631	0.2706	0.3888	0.0441*	
H131	0.3326	0.2659	0.4287	0.0566*	
H141	0.5300	0.3272	0.4883	0.0728*	
H151	0.3864	0.5282	0.5307	0.0784*	
H161	0.0278	0.6652	0.5111	0.0716*	
H171	−0.1695	0.6031	0.4505	0.0557*	
H181	0.2260	0.6167	0.1869	0.0395*	
H182	0.1239	0.4446	0.1778	0.0396*	
H223	0.6719	0.2678	0.0820	0.0701*	
H222	0.8402	0.3475	0.1150	0.0711*	
H221	0.6889	0.4446	0.0854	0.0713*	

### Atomic displacement parameters (Å^2^)

**Table:**

	*U*^11^	*U*^22^	*U*^33^	*U*^12^	*U*^13^	*U*^23^
O1	0.0310 (9)	0.0329 (11)	0.0290 (10)	−0.0035 (9)	−0.0004 (8)	0.0013 (9)
C2	0.0288 (17)	0.0261 (19)	0.0305 (16)	0.0022 (15)	−0.0051 (13)	0.0002 (13)
C3	0.0303 (17)	0.0240 (19)	0.0237 (16)	0.0006 (15)	0.0003 (12)	0.0029 (14)
C4	0.0291 (14)	0.0156 (18)	0.0313 (16)	0.0013 (13)	−0.0048 (14)	0.0002 (14)
C5	0.0299 (15)	0.0260 (18)	0.0316 (16)	−0.0019 (16)	0.0001 (13)	−0.0043 (15)
O6	0.0433 (11)	0.0236 (13)	0.0309 (11)	0.0047 (10)	−0.0042 (9)	−0.0010 (9)
O7	0.0303 (11)	0.0251 (12)	0.0390 (10)	−0.0027 (9)	−0.0010 (8)	−0.0005 (9)
C8	0.0280 (17)	0.028 (2)	0.0287 (18)	−0.0011 (17)	−0.0022 (15)	−0.0061 (16)
O9	0.0453 (12)	0.0285 (12)	0.0417 (12)	0.0080 (11)	0.0038 (10)	0.0052 (10)
N10	0.0378 (13)	0.0288 (15)	0.0334 (13)	0.0078 (13)	−0.0003 (12)	0.0038 (11)
C11	0.0394 (18)	0.044 (2)	0.0322 (17)	−0.0018 (16)	0.0059 (15)	0.0031 (15)
C12	0.0366 (18)	0.042 (2)	0.0282 (17)	−0.0039 (18)	0.0041 (16)	0.0094 (17)
C13	0.048 (2)	0.054 (2)	0.041 (2)	−0.003 (2)	0.0050 (18)	0.0083 (18)
C14	0.046 (2)	0.077 (3)	0.059 (2)	−0.009 (2)	−0.008 (2)	0.021 (2)
C15	0.072 (3)	0.081 (3)	0.042 (2)	−0.022 (3)	−0.010 (2)	0.005 (2)
C16	0.072 (3)	0.066 (3)	0.044 (2)	−0.007 (2)	0.007 (2)	−0.0091 (19)
C17	0.0511 (19)	0.050 (2)	0.0382 (18)	0.0005 (19)	0.0049 (18)	0.0023 (17)
C18	0.0340 (16)	0.032 (2)	0.0346 (17)	−0.0018 (15)	0.0013 (14)	−0.0018 (15)
N19	0.0367 (13)	0.0319 (16)	0.0318 (13)	−0.0115 (13)	0.0074 (12)	−0.0025 (12)
C20	0.0385 (19)	0.034 (2)	0.0254 (16)	0.0013 (19)	−0.0072 (17)	0.0015 (17)
O21	0.0472 (13)	0.0319 (14)	0.0462 (12)	−0.0103 (11)	0.0017 (11)	−0.0039 (10)
C22	0.052 (2)	0.054 (2)	0.0389 (18)	0.0002 (18)	0.0118 (17)	−0.0006 (16)

### Geometric parameters (Å, °)

**Table:**

O1—C2	1.444 (2)	C12—C13	1.379 (3)
O1—C5	1.445 (3)	C12—C17	1.381 (4)
C2—C3	1.518 (3)	C13—C14	1.379 (4)
C2—H22	0.983	C13—H131	0.948
C2—H21	0.981	C14—C15	1.364 (4)
C3—C4	1.555 (3)	C14—H141	0.941
C3—O6	1.429 (3)	C15—C16	1.373 (4)
C3—C8	1.517 (3)	C15—H151	0.956
C4—C5	1.532 (3)	C16—C17	1.386 (3)
C4—O7	1.420 (2)	C16—H161	0.945
C4—H41	0.988	C17—H171	0.937
C5—C18	1.520 (3)	C18—N19	1.443 (3)
C5—H51	0.993	C18—H181	0.998
O6—H61	0.796	C18—H182	0.968
O7—H71	0.801	N19—C20	1.350 (3)
C8—O9	1.234 (3)	N19—H191	0.879
C8—N10	1.330 (3)	C20—O21	1.232 (3)
N10—C11	1.463 (3)	C20—C22	1.497 (3)
N10—H101	0.836	C22—H223	0.956
C11—C12	1.501 (3)	C22—H222	0.947
C11—H111	0.963	C22—H221	0.945
C11—H112	1.007		
			
C2—O1—C5	104.10 (17)	H111—C11—H112	109.4
O1—C2—C3	103.53 (17)	C11—C12—C13	121.1 (3)
O1—C2—H22	110.7	C11—C12—C17	120.0 (3)
C3—C2—H22	113.4	C13—C12—C17	118.9 (3)
O1—C2—H21	109.3	C12—C13—C14	120.4 (3)
C3—C2—H21	110.6	C12—C13—H131	120.3
H22—C2—H21	109.2	C14—C13—H131	119.3
C2—C3—C4	101.29 (19)	C13—C14—C15	120.7 (3)
C2—C3—O6	109.3 (2)	C13—C14—H141	117.6
C4—C3—O6	111.3 (2)	C15—C14—H141	121.7
C2—C3—C8	114.3 (2)	C14—C15—C16	119.6 (3)
C4—C3—C8	111.0 (2)	C14—C15—H151	119.8
O6—C3—C8	109.4 (2)	C16—C15—H151	120.6
C3—C4—C5	104.63 (19)	C15—C16—C17	120.2 (3)
C3—C4—O7	114.56 (19)	C15—C16—H161	121.4
C5—C4—O7	112.09 (19)	C17—C16—H161	118.3
C3—C4—H41	109.5	C16—C17—C12	120.2 (3)
C5—C4—H41	109.7	C16—C17—H171	120.5
O7—C4—H41	106.4	C12—C17—H171	119.3
C4—C5—O1	105.40 (17)	C5—C18—N19	113.39 (19)
C4—C5—C18	114.6 (2)	C5—C18—H181	110.5
O1—C5—C18	110.66 (19)	N19—C18—H181	107.5
C4—C5—H51	107.7	C5—C18—H182	107.8
O1—C5—H51	110.6	N19—C18—H182	109.3
C18—C5—H51	107.9	H181—C18—H182	108.2
C3—O6—H61	109.1	C18—N19—C20	122.2 (2)
C4—O7—H71	108.0	C18—N19—H191	117.8
C3—C8—O9	120.1 (2)	C20—N19—H191	119.9
C3—C8—N10	115.9 (3)	N19—C20—O21	122.1 (2)
O9—C8—N10	123.8 (2)	N19—C20—C22	115.2 (3)
C8—N10—C11	123.6 (2)	O21—C20—C22	122.7 (3)
C8—N10—H101	117.8	C20—C22—H223	111.9
C11—N10—H101	118.5	C20—C22—H222	111.9
N10—C11—C12	112.9 (2)	H223—C22—H222	110.6
N10—C11—H111	108.9	C20—C22—H221	110.7
C12—C11—H111	108.1	H223—C22—H221	105.3
N10—C11—H112	106.4	H222—C22—H221	106.0
C12—C11—H112	111.2		

### Hydrogen-bond geometry (Å, °)

**Table:**

*D*—H···*A*	*D*—H	H···*A*	*D*···*A*	*D*—H···*A*
O6—H61···O7^i^	0.80	1.95	2.737 (4)	167
O7—H71···O1^ii^	0.80	2.08	2.821 (4)	154
N10—H101···O21^i^	0.84	2.29	3.023 (4)	147
N19—H191···O9^iii^	0.88	2.04	2.861 (4)	155
C2—H22···O21^iii^	0.98	2.45	3.322 (4)	148
C4—H41···O1^iv^	0.99	2.59	3.520 (4)	156
C4—H41···O7^i^	0.99	2.49	3.257 (4)	134

Symmetry codes: (i) −*x*, *y*+1/2, −*z*+1/2; (ii) −*x*+1, *y*−1/2, −*z*+1/2; (iii) −*x*+1, *y*+1/2, −*z*+1/2; (iv) *x*−1, *y*, *z*.
